# Discovery of Novel Anti-prion Compounds Using *In Silico* and *In Vitro* Approaches

**DOI:** 10.1038/srep14944

**Published:** 2015-10-09

**Authors:** Jae Wook Hyeon, Jiwon Choi, Su Yeon Kim, Rajiv Gandhi Govindaraj, Kyu Jam Hwang, Yeong Seon Lee, Seong Soo A. An, Myung Koo Lee, Jong Young Joung, Kyoung Tai No, Jeongmin Lee

**Affiliations:** 1 Division of Zoonoses, Center for Immunology & Pathology, National Institute of Health, Korea Centers for Disease Control & Prevention, Chungcheongbuk-do 363-700, Korea; 2 Bioinformatics & Molecular Design Research Center, Seoul, 120-749, Korea; 3 GachonBioNano Research Institute, Gachon University, Gyeonggi-do 461-701, Korea; 4 College of Pharmacy, Chungbuk National University, Cheongju 361-763, Korea; 5 Department of Biotechnology, Yonsei University, Seoul, 120-749, Korea; 6 Nano/Bio Computational Chemistry Laboratory, Department of Chemistry, Sookmyung Woman’s University, Seoul, 140-742, Korea

## Abstract

Prion diseases are associated with the conformational conversion of the physiological form of cellular prion protein (PrP^C^) to the pathogenic form, PrP^Sc^. Compounds that inhibit this process by blocking conversion to the PrP^Sc^ could provide useful anti-prion therapies. However, no suitable drugs have been identified to date. To identify novel anti-prion compounds, we developed a combined structure- and ligand-based virtual screening system *in silico*. Virtual screening of a 700,000-compound database, followed by cluster analysis, identified 37 compounds with strong interactions with essential hotspot PrP residues identified in a previous study of PrP^C^ interaction with a known anti-prion compound (GN8). These compounds were tested *in vitro* using a multimer detection system, cell-based assays, and surface plasmon resonance. Some compounds effectively reduced PrP^Sc^ levels and one of these compounds also showed a high binding affinity for PrP^C^. These results provide a promising starting point for the development of anti-prion compounds.

Prion diseases are a group of lethal neurodegenerative diseases of humans and animals, including human Creutzfeldt-Jakob disease; bovine spongiform encephalopathy; scrapie in sheep, hamsters, and mice; and chronic wasting diseases in deer^1^,^2^. There are three causes of prion disease: hereditary, sporadic, and acquired by infection. All of these disease types are known to share the same pathogenic mechanism^2^,^3^.

The central event in prion disease pathogenesis is the conversion of the α-helix-rich cellular form of prion protein (PrP^C^) to a misfolded, β-sheet-rich, pathogenic, and infectious conformational isoform (PrP^Sc^), although the detailed structure of PrP^Sc^ is still not fully characterised^1^,^4^,^5^. This conversion initiates a chain replication reaction, where each newly converted PrP^Sc^ molecule interacts with more PrP^C^ molecules, fueling the formation of additional PrP^Sc^^6^,^7^. After this post-translational conversion, PrP^Sc^ aggregates and becomes the detergent-insoluble, partially protease-resistant protein fraction that serves as the marker for prion diseases^8^,^9^. Therefore, stabilization of the native PrP^C^ conformation, without blocking the normal functions of PrP^C^, could reduce the rate of conversion to PrP^Sc^ or even prevent prion disease.

To date, screening has led to the identification of many anti-prion compounds^10^. Several large molecules (pentosanpolysulfate^5^, suramin^11^, amphotericin B^12^, congo red^13^, and dendritic polyamines^14^) and small molecules (bis-acridine^15^, polyphenol, phenothiazine, anti-histamine, statin, and some anti-malarial agents including quinacrine^16^) have been reported to inhibit PrP^Sc^ formation or to reduce the level of PrP^C^. The tyrosine kinase inhibitor, STI571 (Gleevec), cured scrapie-infected cells in a concentration- and time-dependent manner with an IC_50_ below 1 μM, by inducing cellular clearance of PrP^Sc3^. In addition, phenothiazine, statin, and quinacrine provide attractive options because they have been approved by the United States Food and Drug Administration for use in other diseases^7^,^9^. However, these drugs were shown to be ineffective against prion disease in rodents^10^,^17^. The toxicity of anti-prion compounds and their inability to cross the blood-brain barrier has limited their successful application^18^.

In cell culture systems, anti-prion compounds are generally assessed by monitoring the levels of protease-resistant PrP^Sc^ using proteinase K (PK) digestion followed by western blotting. As this screening approach is fairly time-consuming and semi-quantitative, we employed a highly quantitative high-throughput misfolded protein detection assay (multimer detection system; MDS) to screen compounds for anti-prion efficacy. This competition assay uses a magnetic bead-conjugated capture antibody and a horseradish peroxidase (HRP)-conjugated detection antibody, with overlapping epitopes to achieve specific detection of multimers (such as PrP^Sc^), and not monomers (such as PrP^C^). The T2 and 3E7 prion antibodies employed by the MDS recognize amino acids 147–152 and 140–160, respectively, of the PrP sequence^19^.

Although PrP^C^ and PrP^Sc^ usually have identical primary amino acid sequences, it has been shown that the conversion from PrP^C^ to PrP^Sc^ causes a substantial change in the secondary protein structure at various locations, including the factor X-binding site, the hotspot binding site, and the unstructured N-terminal binding site^20^,^21^,^22^. Several computational and biophysical studies have targeted these significant regions and used well-known anti-prion compounds to demonstrate stabilization of the secondary structural changes^23^,^24^. Anti-prion compounds that have been identified by different research groups possess diverse scaffolds and similar inhibitory activities, highlighting the need for clarification of the structure-activity relationship (SAR).

The recent development of structure-based virtual screening supported by docking simulations has facilitated effective *in silico* screening of the interactions between chemical compounds and their target proteins, which can contribute to the identification of a desired activity from a large database of chemicals that are structurally different from known active compounds, reducing the time and cost of identifying chemical hits^25^,^26^. Using the structure of PrP^C^-GN8 (a known anti-prion compound), a 3D pharmacophore model was generated and compounds were docked into the prion hotspot to determine their potential binding mode, which enabled the selection of a small number of molecules for *in vitro* testing. In total, 37 compounds were assessed by MDS assay, in scrapie-infected mouse neuroblastoma N2a (ScN2a), in PrP^C^-overexpressing N2a (L2-2B1) cells, and by surface plasmon resonance (SPR) direct-binding experiments.

## Results

### Virtual Screening

**Figure 1:**
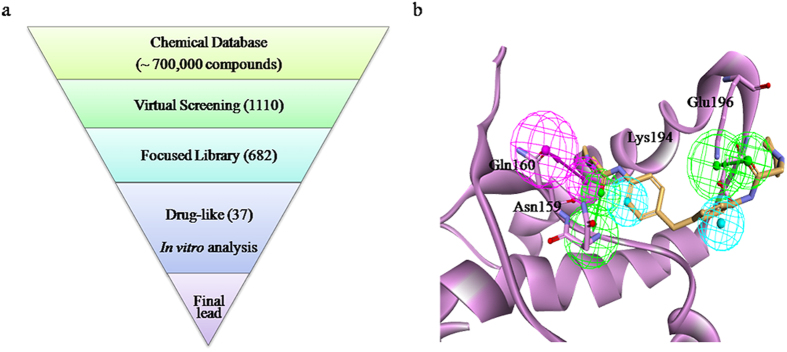
The compound discovery process. (**a**) Work flow for the *in silico* screen and *in vitro* assays. (**b**) The proposed pharmacophore model, showing prion protein in the normal conformation (PrP^C^; pink ribbon representation) mapped with the active anti-prion agent, GN8 (yellow stick representation). The model consists of five features: two hydrophobic elements (cyan), two hydrogen bond acceptors (green), and a hydrogen bond donor (pink).

The overall discovery steps employed in the present study are shown in Fig. 1a. Ten pharmacophore models were generated using the receptor-ligand interaction protocols in Discovery Studio (DS) and the best was chosen using the Genetic Function Approximation (GFA) model (Fig. 1b). To generate the receptor-ligand interaction-based pharmacophore model, a well-defined anti-prion compound (GN8) was docked into the PrP^C^ hotspot (1AG2), as shown in Supplementary Figure 1a^27^,^28^. Although several crystal and nuclear magnetic resonance (NMR) structures are available for PrP^C^, it should be noted that only the NMR of PrP^C^ (1AG2) with GN8 structural details has been thoroughly characterised^23^. Because the pharmacophore model was based on the previously reported NMR structure of PrP^C^-GN8, we expected it to include two important hydrogen bonds from Glu196 and Asn159. As anticipated, the pharmacophore model included five features: the two key hydrogen bonds, two hydrophobic features (H), two hydrogen bond acceptors (HBAs), and one hydrogen bond donor (HBD) (Fig. 1b). To validate this pharmacophore model, we also mapped a previously reported anti-prion compound, GJP49.

Virtual screening of our in-house chemical database with the selected pharmacophore model yielded 1110 compounds. Cluster analysis was performed to filter these compounds, and 682 diverse compounds were selected based on their structural similarities and differences. The compounds were further subjected to visual inspection, resulting in the selection of 37 drug-like compounds for further evaluation.

### PrP^C^ Compound Docking

**Figure 2:**
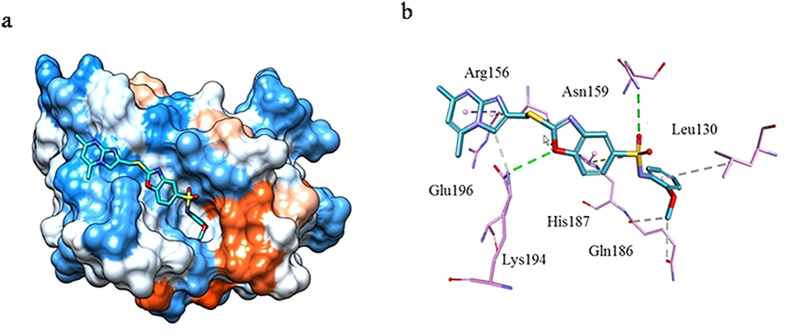
The binding mode and interaction between a compound and prion protein in the normal conformation (PrP^C^). (**a**) The predicted mode of a compound on the surface of PrP^C^, colored to indicate hydrophobicity (blue for the most hydrophilic, to white, to orange-red for the most hydrophobic). (**b**) Close-up view of the interaction between the important PrP^C^ residues (pink sticks) and the compound (cyan stick). Hydrogen bonds are shown as green dashed lines and hydrophobic contacts as pink dashed lines.

The highest binding energy of the 37 hit compounds was measured using the AutoDock score. All compounds were docked into the PrP^C^ hotspot in the same way, with a similar binding mode. Fifty docked conformations were obtained per compound and their best binding energy (kcal/mol) is listed in Table 1. To illustrate the interaction, the binding modes of the PrP^C^-compound interactions are shown in Fig. 2, which illustrates a compound anchored at the PrP^C^ hotspot (Fig. 2a) and interacting with specific amino acids via multiple hydrogen bonds and hydrophobic interactions (Fig. 2b).

### MDS Assay of PrP^Sc^ Inhibition

**Figure 3:**
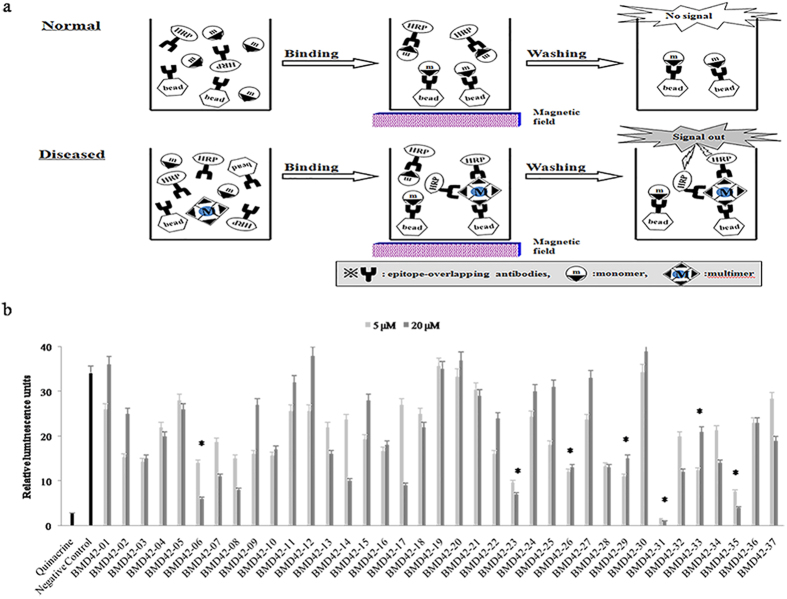
Multimer detection assay. (**a**) The principle of the multimer detection system (monomer: PrP^C^, multimer: PrP^Sc^). (**b**) Luminescence (as a marker of scrapie prion protein [PrP^Sc^] formation) is shown for each of the indicated treatments and concentrations. Values represent the mean of triplicate determinations. Error bars show the standard deviation. The effective reduction of PrP^Sc^ level is indicated as an asterisk. Student’s t-test was used and differences were considered statistically significant at a *P* value of < 0.05.

Our primary *in vitro* screening test employed the MDS enzyme-linked immunosorbent assay (ELISA) to quantify PrP^Sc^ formation. Recombinant PrP was exposed to each of the 37 hit compounds or 1 μM of quinacrine as a positive control. As shown in Fig. 3, quinacrine significantly reduced the formation of PrP^Sc^. The effects of the hit compounds in this assay were not always concentration-dependent. Some compounds actually increased PrP^Sc^ formation at a concentration of 20 μM (BMD42-01, 12, 19, 20, and 30). Twelve compounds (BMD42-03, 06, 07, 08, 10, 16, 23, 26, 28, 29, 31, and 35) exhibited >50% inhibition of PrP^Sc^ formation at both concentrations tested (5 μM and 20 μM). Based on this primary screening, we selected 7 compounds with statistically significant effects (BMD42-06, 23, 26, 29, 31, 33, and 35) for secondary cell culture screening. One of the selected compounds (BMD42-31) showed outstanding inhibitory effects in the MDS assay and even out-performed the positive control, quinacrine.

### Cytotoxicity Assay

**Figure 4:**
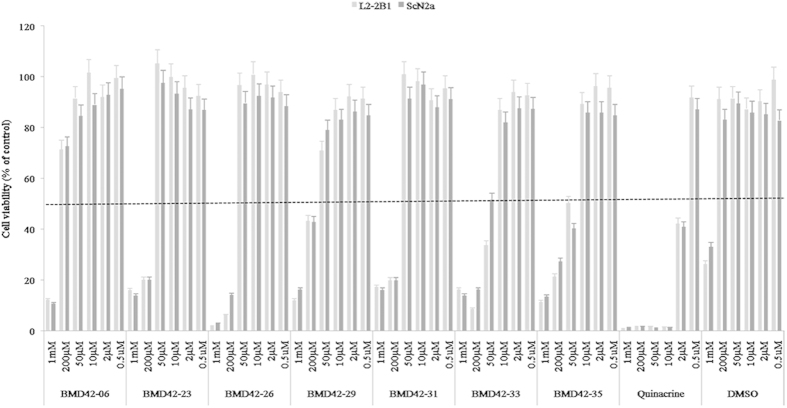
Effect of the compounds on cell viability. Cell viability was determined in the indicated cell lines (L2-2B1 and ScN2a) in the presence of the compounds indicated. The dotted line indicates 50% cytotoxicity. Values represent the mean of three independent experiments and the standard deviation is shown as the error bars.

We used a commercially available cytotoxicity assay to identify suitable treatment concentrations for each compound. Seven compounds were tested at six different concentrations between 0.5 μM and 1 mM (Fig. 4) in ScN2a and L2-2B1 cells. Most of the compounds caused severe cytotoxicity at concentrations above 200 μM. BMD42-06 showed the lowest cytotoxicity of the 7 compounds tested. Concentrations between 0.5 and 30 μM were considered optimal, and concentrations of 5 and 20 μM were thus selected for further study. There were no observable differences in cell viability between the ScN2a and L2-2B1 cell lines. We observed an obvious cytotoxic effect of quinacrine at above 2 μM in both cell lines.

### Compound Effects on PrP^C^ and PrP^Sc^ Propagation in Cultured Cells

**Figure 5:**
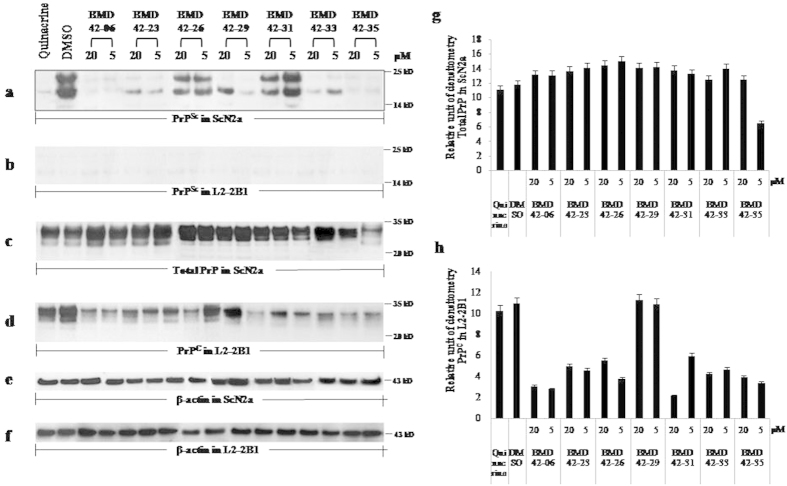
Western immunoblots of proteinase K-digested (**a, b**) and undigested (**c, d**) cell lysates. ScN2a (**a**,**c**) and L2-2B1 (**b**,**d**) cells were treated with the indicated compounds for 6 days. Lane 1, lysates from cells treated with 1 μM quinacrine; lane 2, 0.1% DMSO; subsequent lanes, 20 or 5 μM of the indicated compounds. β-actin immunoblots are shown in ScN2a (**e**) and L2-2B1 (**f**) as protein loading controls. Relative units of densitometry for (**b**,**d**) indicate the volume intensity/mm^2^, relative to the β-actin signal in (**g**,**h**) respectively. Each value represents the mean ± standard deviation; *P* < 0.05. Three independent experiments were performed in duplicate and representative immunoblots are shown. Molecular mass markers are indicated on the right of the immunoblots.

ScN2a cells are infected with the Rocky Mountain Laboratory scrapie prion strain and persistently produce PrP^Sc^. This cell model is widely used to screen anti-prion candidate compounds. We first used a transfected cell line over-expressing PrP^C^ to examine anti-prion effects. This cell line facilitated the observation of changes in the amount of PrP^C^, relative to a normal neuronal cell. Figure 5 shows representative immunoblots for PrP^Sc^ and PrP^C^ or total PrP obtained from ScN2a and L2-2B1 cells exposed to different concentrations of the 7 test compounds, quinacrine (1 μM), or dimethyl sulfoxide (DMSO). Compounds with anti-prion activity would reduce the protein levels. The 7 compounds selected by MDS primary screening revealed a variety of inhibitory effects on PrP^Sc^ levels in ScN2a cells (Fig. 5a). The amount of PrP^Sc^ was concentration-independently reduced by BMD42-06 and 35. The extent of this reduction equaled the effect of quinacrine (Fig. 5a lane 1). BMD42-23, 29 and 33 also reduced PrP^Sc^ levels, whereas BMD42-26 and 31 only produced minor inhibitory effects. However, the reduced levels were not detected in L2-2B1 cells during this study, irrespective of whether the cells were exposed to hit compounds or controls (Fig. 5b). In ScN2a cells, total PrP levels were reduced in the presence of BMD42-35 but were unaffected by quinacrine, DMSO, or the other 6 hit compounds (Fig. 5c,g). In L2-2B1 cells, PrP^C^ was inhibited by 6 compounds, but not by BMD42-29 (Fig 5d,h). Thus, had only ScN2a cells been used, inhibitory effects against PrP^C^ may not have been found for other compounds. Among them, BMD42-35 showed the strongest inhibitory effects in the cell-based assay. Quinacrine completely inhibited the formation of PrP^Sc^, but not PrP^C^, at 1 μM.

### SPR Measurement of Compound-PrP^C^ Binding Affinities

**Figure 6:**
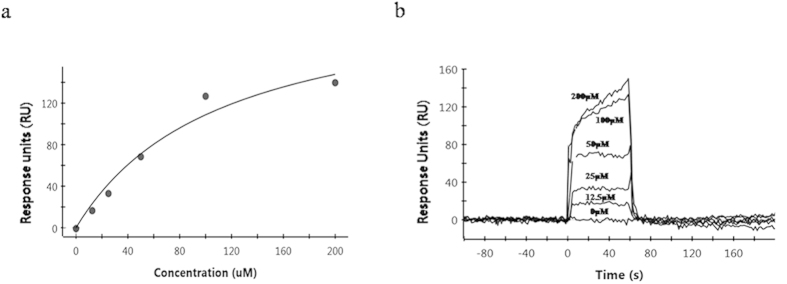
Kinetic analysis of a compound-prion protein (PrP^C^) interaction using surface plasmon resonance. (**a**) The steady state (equilibrium) response units (RU) after fitting are plotted against the concentration of BMD42-29. (**b**) Sensogram curve depicting the interaction between the indicated concentrations of BMD42-29 and sensorchip-immobilized PrP^C^. The equilibrium dissociation rate constant (K_D_) was determined to be 103 μM for this compound.

The 37 hit compounds identified by VS were tested by SPR to quantify their direct PrP^C^ binding ability. Multiple strategies were explored for the immobilization of PrP^C^ to the surface of a high-density sensor chip. Direct measurements of quinacrine binding were repeatedly performed to confirm the stability and function of the coated chips. SPR revealed that the majority of the compounds interacted directly with PrP^C^. The apparent affinities were determined for 23 compounds (Table 1, dissociation constant rate; K_D_). The remaining 14 compounds were tested, but did not bind. These compounds showed consistent results in the MDS assay. Sensorgram curves revealed that similar to previously reported GJP derivatives, our compounds showed single and specific binding to PrP^C^. In particular, BMD42-29 showed rapid association and slow dissociation rates (Fig. 6). This compound showed at least 2-fold tighter binding to PrP^C^ than the other hit compounds. Notably, these findings correlated with those of the computational docking study, where BMD42-29 had the highest AutoDock binding energy (−7.87 kcal/mol) of the hit compounds tested (Table 1). BMD42-35 was found to be effective in the cell-based assay, but did not show binding in the SPR assay.

### Binding Mode and Selectivity

The predicted binding modes for the 37 hit compounds were analyzed to investigate the structural basis of their selectivity. We grouped the compounds by their inhibition efficacy to examine the SAR. Although BMD42-35 was one of the most active compounds in the cell-based assay, it showed no binding in the SPR assay. Several of the other hit compounds showed independent anti-prion activity in cell-based and SPR assays (BMD42-2, 23, 29 and 33). Comparison of the scaffolds of these 4 compounds revealed diverse structural features. Notably, these most active compounds included three sulfonamide compounds (BMD42-23, 29, 33), two thiazole compounds (BMD42-02 and 23) and one benoxazole compound (BMD42-29). The 2-amino thiazole scaffold present in BMD42-23 had previously been widely studied in the context of anti-prion drug discovery and had even been used in a clinical trial for prion disease^29^.

We next investigated the binding mode of each compound with PrP^C^, bearing in mind that two strong hydrogen bonds from Asn159 and Glu196 play important roles in inhibitory activity (Fig. 2). In addition to the high affinity compounds, 10 more compounds (BMD42-31, 26, 16, 03, 04, 10, 22, 07, 34, and 25) were scrutinized. Their chemical structures included amide (BMD42-04 and 22), sulfonamide (BMD42-07 and 34), and pyrazole (BMD42-26) scaffolds. Notably, BMD42-16 and 31 possessed both amide and sulfonamide scaffolds and showed partial efficacy in SPR and MDS assays. BMD42-03, 10, and 25 possessed diverse scaffolds and were less active in all screening tests. In addition, their binding modes predicted a single hydrogen bond and hydrophobic contacts with the hotspot residues of Glu196/Asn159/Lys194/Glu160/Leu130, which may contribute to stabilization of the PrP^C^ structure. In addition, we grouped 7 moderately active compounds (BMD42-17, 30, 19, 24, 36, 14, and 12) and elucidated their binding with hotspot residues. The chemical structures of these compounds confirmed that the presence of sulfonamide and amide scaffolds (BMD42-24 and 14) produced potent anti-prion effects.

To confirm the above findings, we investigated two less active compounds, BMD42-01 and 05 (Supplementary Fig. 2). These compounds had no hydrogen bonding or hydrophobic interactions with key hotspot residues, as their structures differed from those of the most active compounds. In contrast, one of the most active compounds (BMD42-29) showed strong hydrogen bonding at the helix regions of Asn159 and Glu196, and hydrophobic interactions with Leu130 and Arg156. These findings emphasized the importance of these hydrogen bonds and hydrophobic interactions for efficient anti-prion effects. Compounds lacking interaction with Asn159 and Glu196 were unable to inhibit PrP^Sc^ formation (Supplementary Fig. 2).

## Discussion

Research aiming to develop treatments for prion diseases has led to the identification of a range of compounds such as quinacrine and doxycycline that can selectively block conversion of PrP^C^ to PrP^Sc^^30^. However, these compounds have not provided useful treatments for patients. To discover and validate novel non-toxic and effective anti-prion drugs, we studied a structurally diverse series of compounds with anti-prion activity using an *in silico* approach. Use of the structures of PrP^C^-hit compound complexes enabled analysis of compound localization at the hotspot sites and interactions with key residues in the previously determined NMR PrP^C^-GN8 complex.

The three most effective strategies for discovering anti-prion compounds include screening for inhibitory effects on PrP^Sc^ accumulation, inactivation of endogenous PrP^C^ (as the substrate for prion conversion), and the enhancement of PrP^Sc^ degradation^31^. Inhibition of *in vitro* PrP^Sc^ accumulation represents a primary target for prion disease therapy^16^,^32^.

Expression of PrP^c^ in host neurons is required for PrP^Sc^ replication and disease progress. As such, persistently infected cell lines acting as a host for PrP^Sc^ are frequently used to study prion-related cellular processes, and also to screen for effective anti-prion compounds^33^. ScN2a cells have been used extensively as a relevant model for the study of prion diseases^34^. In the present study, considerable reductions in PrP^C^ levels were only observed in L2-2B1 cells, possibly because the cells had higher initial levels of PrP^C^. The L2-2B1 cell line was therefore very useful for analyzing this aspect of the compounds’ activities. Quinacrine was cytotoxic in both cell lines at concentrations above 2 μM. This result was consistent with a previous study in which cytotoxicity was tested in the presence of 0.02–200 μM quinacrine, and the optimal concentration found to inhibit PrP^Sc^ formation was below 4 μM^7^.

We performed a highly quantitative and precise protein misfolding detection assay for the primary screening of anti-prion compound efficacy. The results of this MDS assay facilitated the selection of 7 potent hit compounds (BMD42-06, 23, 26, 29, 31, 33, and 35). Although BMD42-31 was more effective than quinacrine in the MDS assay, it was excluded during our secondary cell-based screening as it produced only a minor reduction in PrP^Sc^ level, although the PrP^C^ level was reduced at 20 μM. Chemical modification of this compound may increase its efficacy.

The MDS assay results appeared to indicate that BMD42-06, 23, 26, 29, 31, 33, and 35 conformationally stabilized PrP^C^ by reducing its aggregation. BMD42-06, 23, 26, 31, 33, and 35 also decreased the level of PrP^C^. The mechanisms underlying this effect may involve PrP^C^ consumption as a substrate for aggregation; a reduction in the PrP mRNA level; or protein degradation caused by compound-PrP^C^ binding. The results indicated that in ScN2a cells, BMD42-06, 23, 29, 33, and 35 reduced the amount of PrP^Sc^, either by interfering with the conformational interaction between PrP^C^ and PrP^Sc^, or by interacting directly with PrP^Sc^.

Recently, Kamatari and co-workers classified anti-prion compounds according to four potential molecular mechanisms of action: (i) specific conformational stabilization of PrP^C^; (ii) nonspecific stabilization, including interference with the conformational interaction between PrP^C^ and PrP^Sc^, in addition to hotspot binding; (iii) promotion of PrP^C^ aggregation and precipitation, thus reducing the amount available for conversion to PrP^Sc^; or (iv) interactions with molecules other than PrP^C^, such as PrP^Sc^ or membrane proteins^35^. The compounds identified in the present study showed multiple mechanisms of action, based on their effects on PrP^C^ aggregation, reduction, and PrP^Sc^ propagation, as well as their binding affinities. Placing our results in the context of Kamatari’s classification, BMD42-29 may act by specific stabilization of PrP^C^, as evidenced by its strong binding affinity. BMD42-06, 23, 29, 33, and 35 may act by nonspecific blockade of PrP^C^ conversion to PrP^Sc^, or by interacting with PrP^Sc^, as evidenced by their weak binding affinities and the observed reduction in the level of PrP^Sc^ or PrP^C^ aggregate. BMD42-06, 23, 26, 31, 33, and 35 may act by reducing the amount of PrP^C^ by reducing PrP expression, or stimulating PrP degradation. Further mechanistic studies are required to investigate exactly how these compounds alter PrP^C^ levels.

Our results suggest that BMD42-29 is an optimal compound, exhibiting PrP^Sc^ inhibition and a stronger binding affinity than other anti-prion compounds reported to date. However, it did not produce a marked reduction in PrP^C^ levels, possibly indicating that it stabilized PrP^C^ and inhibited its pathological conformation change to PrP^Sc^. BMD42-35 produced the highest decrease in PrP^Sc^ and PrP^C^ levels, although it showed a lower binding affinity, which suggests that BMD42-35 acts by either stabilizing or eliminating PrP^C^ and may control PrP^C^ non-specifically. This contradictory result may relate to problems with SPR assays reported by earlier studies, including the unlimited increase in ProteOnGLH sensor responses, low solubility of compounds, and slow binding kinetics^36^.

It has been proposed that the hydrogen bonds from stand S1 and helix B may prevent the conversion of PrP^C^ to PrP^Sc^^21^. Collectively, our compounds showed strong hydrogen bonds at Asn159 (stand S1) and Glu196 (helix B) within PrP^C^. The most active compounds occupied the hydrophobic area highlighted in the pharmacophore model generated from the PrP^C^-GN8NMR structure. BMD42-29, which was active in both cell-based and SPR-based assays, and BMD42-35, which was most active in the cell-based assay, both interacted with conserved PrP^C^ hotspot residues, indicating the importance of the two hydrogen bonds and the hydrophobic environment; this was predicted by the pharmacophore model.

Further elucidation of the mechanisms of action of BMD42-29 and BMD42-35 will provide unique tools to study the mechanism of prion replication. The approach used in the present study may provide *in vitro* screening data that are more highly predictive of *in vivo* activity, contributing to the rational design of novel and effective anti-prion treatments.

## Methods

### Pharmacophore Development

The protein data bank NMR structure of PrP^C^ (1AG2) was used as the template for pharmacophore generation^37^. DS software was used to map the active site of PrP^C^ and critical residues were identified based on the PrP^C^-GN8 NMR data^10^. Subsequently, the receptor-ligand pharmacophore generation protocols were used to generate a quantitative model of PrP^C^. The protocols resulted in 10 3D pharmacophore hypotheses and the highest ranked of these was selected.

### Virtual Screening

The 3D pharmacophore hypothesis was used to extract chemical compounds from our in-house database of 700,000 compounds. The screening processes were performed using the *best flexible conformation search* method in DS, with standard settings.

### Molecular Docking

Molecular docking calculations were performed using the AutoDock tool. To validate the AutoDock, we docked the known anti-prion compounds, GN8 and GJP49, into the PrP^C^ hotspot (1AG2). A grid of 63, 57, and 53 points in the x, y, and z directions was constructed on the center of the PrP^C^ hotspot mass. A default setting grid spacing of 0.375 Å and a distance-dependent function of the dielectric constant were used for the energetic map calculations. The docked compounds were subjected to 50 runs of the AutoDock Lamarckian genetic algorithm, with 500,000 steps of energy evaluation and default values for the other parameters. Cluster analysis was performed on the results using 1.0 Å. The docking pose analysis was conducted for the first pose of the most populated cluster in the AutoDock output.

### Compounds and Cell lines

BMD42-01–06, 21–24, 27–30, 32–34, 36, and 37 compounds were purchased from Enamine. BMD42-07 and 08 were purchased from ASINEX. BMD42-09–11 were purchased from Life Chemicals. BMD42-12–15 were purchased from Chembridge. BMD42-16 and 26 were purchased from ChemDiv. BMD42-17 was purchased from Vista M labs. BMD42-18–19 and 35 were purchased from Princeton Biomolecular Research. BMD42-20 was purchased from Synthon-lab. BMD42-25 was purchased from Sigma Aldrich. BMD42-31 was purchased from Uorsy. The compounds were dissolved in DMSO, diluted to produce a 50 mM stock solution, and stored at –20°C. ScN2a cells were derived from N2a cells obtained from the ATCC and were generously provided by Dr. Ryu, Hanyang University, Korea. The L2-2B1 subclone cell line overexpressing PrP^C^ derived from N2a was established by Dr. Kim, Korean National Institute of Health.

### MDS Assay

The MDS assay kit was supplied by People Bio Inc. and performed according to the instructions, with minor revision^19^. Briefly, each compound was pre-treated with the reaction buffer containing 50 ng of recombinant PrP, 1% Triton X-100, 10% Blockace and Tris-buffered saline containing 0.1% (vol/vol) Tween 20 (TBST) in 2 mL screw cap tubes. DMSO (0.1% vol/vol) was used as a negative control and quinacrine (1 μM) (Sigma Aldrich, Q3251) was the positive control. The mixture was incubated with continuous shaking for 3 h at 37 °C. The 3E7 PrP antibody (2 μg), conjugated to magnetic beads, and the HRP-conjugated PrP T2 antibody (8 μg) were added to the pre-incubated mixture. After incubation for 1 h under the same conditions, the beads were separated and washed three times with TBST using a magnetic particle concentrator (Invitrogen, 120.20D). The assay signal was developed by adding Supersignal ELISA pico chemiluminescence substrate (Pierce, 37070) and quantified using a VICTOR3 microplate reader (Perkin Elmer, 1420-032).

### Cell Culture and Treatments

ScN2a and L2-2B1 cells were seeded in six-well plates (2 × 10^5^ cells/well) and incubated in Opti-MEM (Gibco, 31985) containing 10% fetal bovine serum, 1% penicillin-streptomycin (Gibco, 15140) and 2 mM l-glutamine (Gibco, 25030) with the indicated concentrations of the compounds in 5% CO_2_ at 37 °C. The stock compound solutions (50 mM) were diluted in Opti-MEM. Control cell cultures were treated with DMSO only (0.1% v/v). Each compound was tested in duplicate in three independent experiments.

### Lysis and PK Digestion

Cells were rinsed once in phosphate-buffered saline (PBS) and then incubated with 0.1% (vol/vol) trypsin-EDTA (Gibco, 25300) for 1 min at room temperature. The detached cells were centrifuged at 4000 × g for 10 min at 4 °C and rinsed once with PBS. Cells were lysed with ice-cold lysis buffer (10 mM EDTA, 10 mM Tris, pH 8.0, 100 mM NaCl, 0.5% [wt/vol] Nonidet P-40, and 0.5% [wt/vol] sodium deoxycholate). Two freeze-thaw cycles in liquid nitrogen were performed, followed by sonication at an amplitude of 30%. The total protein concentration was adjusted to 1 mg/mL.

For PrP^Sc^ detection, cell lysates were digested with PK (20 μg/mL) (Merck, 70663) for 60 min at 37 °C. The reaction was stopped with 2 mM Pefabloc (Roche, 11429876001), and the lysates were centrifuged for 90 min at 20,000 × *g* at 4 °C. Pellets were resuspended in sodium dodecyl sulfate (SDS) sample buffer (125 mM Tris-HCl, pH 6.8, 5% [vol/vol] glycerol, 6 mM EDTA, 5% [wt/vol] SDS, 0.04% [vol/vol] bromophenol blue, and 12.5% [vol/vol] β-mercaptoethanol). For PrP^C^ detection, cell lysates were treated with 2 mM Pefabloc only to achieve the same conditions, and then resuspended in SDS sample buffer.

### Western Immunoblotting

Protein samples were separated by SDS-polyacrylaminde gel electrophoresis for 2 h at 150 V at 4 °C, using 12% gels. Proteins were then transferred to a polyvinylidenefluoride membrane using the i-Blot system (Invitrogen) for 7 min. The membrane was blocked for 1 h at room temperature in 5% (wt/vol) skim milk in PBS containing 0.1% (vol/vol) Tween 20 (PBST) and then incubated overnight at 4 °C with the PrP antibody, SAF-32 (1:200) (Cayman Chemicals, 189720) diluted in 10% (vol/vol) blocking buffer. After washing with PBST, the membrane was incubated for 1 h at room temperature with HRP-conjugated anti-mouse IgG (1:2,000) (DAKO, P0260). The signals were visualized using ECL (Elpis Biotech, EBP-1073) and the protein bands were scanned by Image Scanner III (GE). The relative band densities are shown as the volume intensity/mm^2^, relative to the β-actin band density. Blots used for PrP^C^ detection were stripped and then re-probed using a β-actin antibody (1:5000) (Cell Signaling, 4970).

### Cytotoxicity Assay

Cytotoxicity was evaluated using a commercial kit (CellTiter 96 Non-Radioactive Cell Proliferation Assay, Promega, G4001), following the manufacturer’s instructions. ScN2a and L2-2B1 cell suspensions (5,000 cells) were plated into each well of a 96-well plate. After adding the compounds at six different concentrations between 0.5 μM and 1 mM or the control, the cells were incubated for 6 days. Dye solution was then added to each well and incubated for up to 4 h. Solubilization solution/stop mix was then added and cell viability was measured using an ELISA plate reader (Bio-Rad) at a wavelength of 570 nm. Cytotoxicity was expressed as a percentage of the signal observed in untreated control cells.

### SPR Analysis

SPR was conducted using the ProteOn XPR36 protein interaction array system (Bio-Rad Laboratories). PrP was amine-coupled on the ProteOn GLH sensor chip. ProteOn running buffer (PBS, pH 7.4, with 0.05% Tween 20) was used at a flow rate of 100 μL/min. The test compounds (200 μL) were injected at six different concentrations (0 μM, 12.5 μM, 25 μM, 50 μM, 100 μM, and 200 μM). Data were analyzed by ProteOn Manager Software 2.0 using the standard Langmuir models for fitting kinetic data. A high-affinity interaction was characterized by a low K_D_, rapid recognition and binding (rapid ‘on rate’ or high K_a_), and stable complex formation (slow ‘off rate’ or low K_d_), in accordance with the equation, K_D_ = K_d_/K_a_.

### Statistical analysis

Each experiment was repeated a minimum of three times. The one-way analysis of variance with the Tukey-Kramer procedure was used. Differences were considered as significant at *P* < 0.05.

## Additional Information

**How to cite this article**: Hyeon, J. W. *et al.* Discovery of Novel Anti-prion Compounds Using *In Silico* and *In Vitro* Approaches. *Sci. Rep.* **5**, 14944; doi: 10.1038/srep14944 (2015).

## Supplementary Material

Supplementary Information

## Figures and Tables

**Table 1:** Binding Parameters.

Compound	Ka, M^−1^ sec^−1^	Kd, M^−1^ sec^−1^	KD, μM	AutoDockBinding Energy, kcal/mol
**BMD42-01**				−4.4
**BMD42-02**	6.18E + 01	1.63E-03	26.4	−5.71
**BMD42-03**	2.25E + 01	4.26E-03	189	−5.32
**BMD42-04**	2.76E + 03	5.99E-01	217	−5.32
**BMD42-05**				−6.2
**BMD42-06**	2.44E + 06	2.46E + 03	1010	−6.65
**BMD42-07**	1.22E + 03	6.83E-01	559	−6.21
**BMD42-08**				−5.64
**BMD42-09**				−5.86
**BMD42-10**	1.19E + 03	5.25E-01	442	−6.08
**BMD42-11**				−5.82
**BMD42-12**	2.15E + 02	2.15E-01	999	−6.52
**BMD42-13**				−4.76
**BMD42-14**	9.91E + 02	4.39E-01	443	−6.88
**BMD42-15**				−6.56
**BMD42-16**	5.98E + 01	3.37E-03	56.3	−6.35
**BMD42-17**	3.64E + 01	5.67E-03	155	−6.91
**BMD42-18**				−6.34
**BMD42-19**	5.53E + 04	1.08E + 01	195	−6.53
**BMD42-20**				−5.9
**BMD42-21**				−5.04
**BMD42-22**	1.81E + 02	8.15E-02	451	−6.98
**BMD42-23**	2.36E + 01	2.68E-03	114	−7.63
**BMD42-24**	2.19E + 03	5.22E-01	239	−5.86
**BMD42-25**	2.02E + 05	3.31E + 02	1640	−5.67
**BMD42-26**	2.62E + 04	1.59E + 01	606	−5.5
**BMD42-27**				−6.51
**BMD42-28**				−5.97
**BMD42-29**	4.07E + 04	8.76E-01	21.5	−7.87
**BMD42-30**	1.31E + 04	2.49E + 00	191	−5.09
**BMD42-31**	1.03E + 05	1.22E + 01	331	−5.75
**BMD42-32**				−6.73
**BMD42-33**	1.30E + 01	8.03E-03	465	−5.8
**BMD42-34**	1.22E + 03	6.83E-01	559	−6.88
**BMD42-35**				−5.7
**BMD42-36**	2.14E + 03	8.43E-01	394	−6.75
**BMD42-37**				−6.9
**Quinacrine**	6.51E + 01	4.26E-03	30.4	−6.48

K_a_, K_d_, and K_D_ denote association rate, dissociation rate, and dissociation constant rate, respectively. Blanks indicate unbinding.
