# Supplementary material for: Discovery of Novel Anti-prion Compounds Using In Silico and In Vitro Approaches
Source: Sci Rep. 2015 Oct 9;5:14944. doi: 10.1038/srep14944 (PMC4598813; doi:10.1038/srep14944)

Discovery of Novel Anti-prion Compounds Using *In Silico* and *In Vitro* Approaches

Jae Wook Hyeon[1+], Jiwon Choi[2+], Su Yeon Kim[1], Rajiv Gandhi Govindaraj[2], Kyu Jam Hwang[1], Yeong Seon Lee[1], Seong Soo A. An[3], Myung Koo Lee[4], Jong Young Joung[6], Kyoung Tai No[2,5*] & Jeongmin Lee[1*]

[1]Division of Zoonoses, Center for Immunology & Pathology, National Institute of Health, Korea Centers for Disease Control & Prevention, Chungcheongbuk-do 363-700, Korea

[2]Bioinformatics & Molecular Design Research Center, Seoul, 120-749, Korea

[3]GachonBioNano Research Institute, Gachon University, Gyeonggi-do 461-701, Korea

[4]College of Pharmacy, Chungbuk National University, Cheongju 361-763, Korea

[5]Department of Biotechnology, Yonsei University, Seoul, 120-749, Korea

[6]Nano/Bio Computational Chemistry Laboratory, Department of Chemistry, Sookmyung Woman's University, Seoul, 140-742, Korea

[+]Co-first authors

[*]Co-corresponding authors

JeongminLee

Email: jeongminlee@korea.kr

Tel: +82-43-719-8462

Fax: +82-43-719-8489

Postal address: Division of Zoonoses, Center for Immunology & Pathology, National Institute of Health, Korea Centers for Disease Control & Prevention, Chungcheongbuk-do 363-700, Korea

Kyoung Tai No

Email: ktno@bmdrc.org

Tel: +82-2-393-9550

Fax: +82-2-393-9554

Postal address: Bioinformatics & Molecular Design Research Center, Seoul, 120-749, Korea

Department of Biotechnology, Yonsei University, Seoul, 120-749, Korea

Supplementary Figure Legends

Supplementary Figure 1: Benchmark docking of reported anti-prion agents with normal prion protein (PrP^C). (a) Ribbon diagram of PrP^C with GN8. Interacting PrP^C residues are shown as stick representations. (b) Superimposed binding pose of GN8 and GJP49, which are shown as gold and green sticks on the PrP^C surface. The amino acid residues of PrP^C interacting with the compounds are labelled and key residues are circled.

Supplementary Figure 2: Docking poses of the inactive compounds identified in this study with normal prion protein (PrP^C). Predicted binding mode of (a) BMD42-01 and (c) BMD42-05 in the PrP^C binding site. Interacting PrP^C residues are shown as pink sticks, whereas PrP^C and BMD42-01 are shown as a pink ribbon and purple stick representation, respectively. Surface structure representation of PrP^C with (b) BMD42-01 and (d) BMD42-05 in the binding site. The PrP^C molecular surface is colored by hydrophobicity. The AutoDock binding energy value is provided.

Supplementary figure 1

a

b

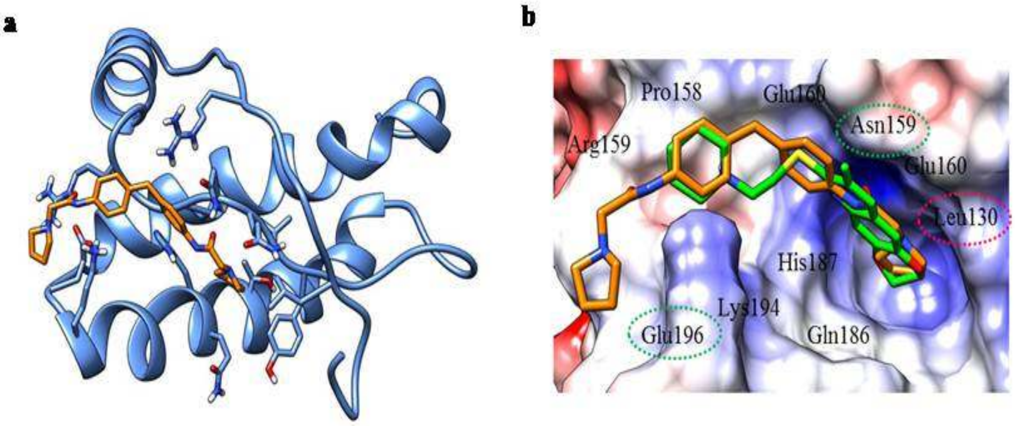

PrPc-GN8

GJ49-GN8

Supplementary figure 2

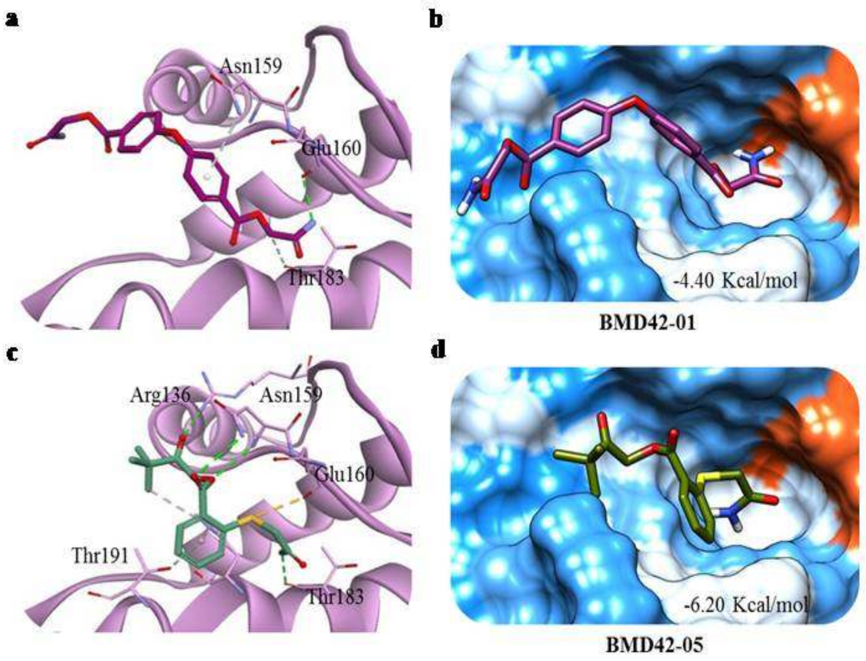

Supplement: Supplementary Information [file srep14944-s1.pdf]
